# Measuring Vitamin D Status in Chronic Inflammatory Disorders: How does Chronic Inflammation Affect the Reliability of Vitamin D Metabolites in Patients with IBD?

**DOI:** 10.3390/jcm9020547

**Published:** 2020-02-17

**Authors:** Aysegül Aksan, Dilem Tugal, Nathalena Hein, Katharina Boettger, Yurani Caicedo-Zea, Ina Diehl, Claudia Schumann, Franz-Paul Armbruster, Jürgen Stein

**Affiliations:** 1 Interdisciplinary Crohn Colitis Center Rhein-Main, Schifferstr. 59, 60594 Frankfurt am Main, Germany; ayseguel.aksan@ernaehrung.uni-giessen.de (A.A.); dilemtugal@hacettepe.edu.tr (D.T.); boettger.katharina@gmail.com (K.B.); 2 Institute of Pharmaceutical Chemistry, Goethe University, 60438 Frankfurt am Main, Germany; 3 Institute of Nutritional Sciences, Justus-Liebig University, 35392 Giessen, Germany; 4 Faculty of Health Sciences, Hacettepe University, 06100 Sihhiye, Ankara, Turkey; 5 DGD Clinics Sachsenhausen, 60594 Frankfurt am Main, Germany; hein_n@web.de; 6 Immundiagnostik AG, 64625 Bensheim, Germany; yurani.caicedo-zea@immundiagnostik.com (Y.C.-Z.); labor3@immundiagnostik.com (I.D.); vitamind@immundiagnostik.com (C.S.); info@immundiagnostik.com (F.-P.A.)

**Keywords:** Vitamin D, vitamin D deficiency, vitamin D metabolites, biomarker, chronic inflammation, inflammatory bowel disease

## Abstract

Evidence gained from recent studies has generated increasing interest in the role of vitamin D in extraskeletal functions such as inflammation and immunoregulation. Although vitamin D deficiency has been implicated in the pathophysiology of inflammatory diseases including inflammatory bowel disease (IBD), evidence as to whether vitamin D supplementation may cure or prevent chronic disease is inconsistent. Since 25OH-vitamin D (25OHD) has been suggested to be an acute-phase protein, its utility as a vitamin D status marker is therefore questionable. In this study, possible interactions of vitamin D and inflammation were studied in 188 patients with IBD, with high-sensitivity C-reactive protein (hsCRP) levels ≥ 5 mg/dL and/or fecal calprotectin ≥ 250 µg/g defined as biochemical evidence of inflammatory activity. Levels of 25OHD and vitamin D-binding protein (VDBP) were determined by ELISA, and 1,25-dihydroxyvitamin D (1,25OHD) and dihydroxycholecalciferol (24,25OHD) by LC-MS/MS. Free and bioavailable vitamin D levels were calculated with the validated formula of Bikle. Serum 1,25OH2D and vitamin D binding protein (VDBP) levels were shown to differ between the inflammatory and noninflammatory groups: patients with inflammatory disease activity had significantly higher serum concentrations of 1,25OH2D (35.0 (16.4–67.3) vs. 18.5 (1.2–51.0) pg/mL, *p* < 0.001) and VDBP (351.2 (252.2–530.6) vs. 330.8 (183.5–560.3) mg/dL, *p* < 0.05) than patients without active inflammation. Serum 24,25OH2D levels were negatively correlated with erythrocyte sedimentation rate (ESR) (−0.155, *p* = 0.049) while concentrations of serum 1,25OH2D correlated positively with hsCRP (0.157, *p* = 0.036). Correlations with serum VDBP levels were found for ESR (0.150, *p* = 0.049), transferrin (0.160, *p* = 0.037) and hsCRP (0.261, *p* < 0.001). Levels of serum free and bioavailable 25OHD showed a negative correlation with ESR (−0.165, *p* = 0.031, −0.205, *p* < 0.001, respectively) and hsCRP (−0.164, *p* = 0.032, −0.208, *p* < 0.001 respectively), and a moderate negative correlation with fecal calprotectin (−0.377, *p* = 0.028, −0.409, *p* < 0.016, respectively). Serum total 25OHD concentration was the only vitamin D parameter found to have no specific correlation with any of the inflammatory markers. According to these results, the traditional parameter, total 25OHD, still appears to be the best marker of vitamin D status in patients with inflammatory bowel disease regardless of the presence of inflammation.

## 1. Introduction

For more than a century, vitamin D has primarily been known for its favorable effects on calcium and bone metabolism [1]. However, in the early 1980s, the vitamin D receptor (VDR) was detected in over 30 different types of human tissue such as peripheral blood monocytes and leukocytes, antigen-presenting cells and activated CD4+ and CD8+ T cells [2,3,4]. More recently, 1,25-dihydroxyvitamin D (1,25OH2D) has been shown to suppress the proliferation of Th1 and Th17 cells and stimulate T regulatory cell activity [5]. Vitamin D also serves to inhibit the demarcation of monocytes to dendritic cells, reducing the availability of antigen-presenting cells for T cell activation [6,7,8]. Over the past few years, increasing numbers of studies have shed light on the role of vitamin D in extraskeletal functions. Furthermore, vitamin D has been characterized as a natural immune modulator [9]. Mounting evidence suggests that vitamin D may play an important pathophysiological role in the conditions associated with immune system dysfunction such as multiple sclerosis, rheumatoid arthritis, insulin-dependent diabetes mellitus and inflammatory bowel disease (IBD) [10,11].

IBD is characterized by chronic relapsing and remitting inflammatory disease of the gastrointestinal tract [12,13], and is currently regarded as incurable. While its exact pathogenesis is still unclear, studies have shown IBD to be associated with genetic predisposition, combined with a complex variety of environmental influences (e.g., diet, smoking, sleep), in addition to microbial and immune factors [14,15,16]. Vitamin D deficiency is known to occur with increased prevalence in patients with Crohn’s disease (CD) and ulcerative colitis (UC), the two predominant pathophysiological manifestations of IBD [17,18]. However, it has yet to be proven whether inadequate vitamin D status acts as a trigger or propagator of inflammation in IBD, whether the deficiency arises purely as a consequence of chronic inflammatory activity or whether there may be some aspects of both. In murine models, outcomes of experimental colitis have been shown to be decisively influenced by vitamin D through VDR signaling [17,19]. This suggests that vitamin D-mediated regulation of T cell development and function may play a crucial role in determining the type and extent of immune response, either encouraging or averting autoimmunity. Whether it is a cause or a consequence, vitamin D deficiency/insufficiency, together with long-term illness and repeated use of corticosteroids, contributes to a significantly increased risk of bone loss and fractures in patients with IBD [20]. Studies have also demonstrated poor vitamin D status to be an independent risk factor for the incidence of colorectal cancer in individuals with IBD [21]. Observational data confirmed low levels of vitamin D to be associated with increased concentrations of hepcidin and thus with anemia, one of the most prevalent extraintestinal complications of IBD [22]. Additionally, vitamin D insufficiency/deficiency has been shown to be associated with poor health-related quality of life (HRQOL) outcomes [23]. Thus, it is important to diagnose, monitor and treat vitamin D deficiency/insufficiency in IBD patients.

Traditionally, the determination of vitamin D status is based on concentrations of circulating levels of total 25-hydroxyvitamin D (25OHD) [24,25], and clinical recommendations for vitamin D sufficiency are based on this parameter [26]. Of all vitamin D metabolites, however, 25OHD is not the most biologically active, requiring further metabolization to 1,25OH2D before it can attain its full biologic capacity [24]. Thus, other vitamin D metabolites, such as 1,25OH2D and dihydroxycholecalciferol (24,25OH2D), are also of potential interest. Furthermore, recent data indicate that other metabolites or analytes of vitamin D may offer important additional insights into vitamin D status, and thus into the role of the vitamin in human health, since concentrations of these substances may not be mirrored by total 25OHD levels [27]. In particular, total 25OHD, vitamin D binding protein (VDBP) and bioavailable and free 25OHD are thought to be promising potential (supplementary) markers. Recently, Strisciuglio et al. [28] published data from a pediatric population with IBD, albeit a relatively small sample, showing that despite low levels of total 25OHD, concentrations of free 25OHD were normal or increased. In addition, whereas a significant direct correlation was identified between free 25OHD and disease activity indices, no similar relationship was found between total 25OHD and serological markers of inflammation (CRP, fecal calprotectin). Ghaly et al. [29] found levels of VDBP, but not total, free or bioavailable vitamin D, to be positively associated with risk of disease flare in patients with Crohn’s disease. There is also some evidence showing that, compared to total 25OHD, bioavailable or free 25OHD may be superior markers of vitamin D status [27,30]. Additionally, either as a cause or as a consequence, low 25OHD levels have been proposed to be related to inflammatory status and/or disease activity in patients with IBD [31,32,33], and data from a number of studies suggest that 25OHD may be an acute-phase reactant [34,35,36,37]. Therefore, the addition of inflammation into this complex picture makes it even more challenging to identify and quantify vitamin D insufficiency. The aim of this study was to explore possible interactions of vitamin D and inflammation in patients with IBD from this perspective and to look for an accurate method of measuring and defining vitamin D status in the context of chronic disease-related inflammation.

## 2. Methods

This study was conducted as a cross-sectional prospective study between January and May 2019 in adult patients previously diagnosed with IBD, according to the guidelines laid down in the Declaration of Helsinki. All procedures involving human participants were approved by the local ethics committee, the Landesärztekammer Hessen (FF 33/2019). All participants gave written informed consent confirming awareness of the investigative nature of the study, possible associated risks and data protection measures.

### 2.1. Study Population

Patients aged 18–65 years consecutively attending the Interdisciplinary Crohn Colitis Center Rhein-Main, Frankfurt am Main, Germany, who had been previously diagnosed with IBD according to standard clinical, radiological and pathological criteria [38,39], were invited to participate in the study. All participants were given detailed information describing study procedures, including data protection measures. Individuals who gave written informed consent and were found eligible were enrolled into the study. Study exclusion criteria were pregnancy, osteoporosis/fracture, granuloma-forming disorders, chronic kidney disease, hepatic failure, cancer, coronary heart disease and diabetes. Also excluded were patients who had used vitamin D or calcium supplements in the previous six months.

### 2.2. Study Design

At enrolment, demographic data, disease characteristics details of ongoing treatment and a recent complete blood count were obtained and documented from the clinic’s medical records. Blood samples were collected on the day after enrolment between 08:00 and 10:00 h, and serum samples were stored at −30 °C until analysis. 

Serum total 25OHD concentrations were measured by direct competitive enzyme-linked immunosorbent assay (ELISA) using the IDK 25OHD immunoassay kit (Immundiagnostik, Bensheim, Germany) and expressed in ng/mL. Vitamin D status of the participants was classified according to the recommendations of the Endocrine Society, with sufficiency defined as 30–100 ng/mL, insufficiency as 21–29 ng/mL and deficiency as < 20 ng/mL [40]. Measurement of circulating serum VDBP concentration was carried out with ELISA using the IDK human vitamin D binding protein immunoassay kit (Immundiagnostik, Bensheim, Germany) according to the manufacturer’s instructions. All measurements were repeated in all serum samples. Serum albumin, transferrin and high sensitivity C-reactive protein (hsCRP) levels and fecal calprotectin concentrations were analyzed according to the guidelines of the German United Society for Clinical Chemistry and Laboratory Medicine (Deutsche Vereinte Gesellschaft für Klinische Chemie und Laboratoriumsmedizin, DGKL) at a local reference laboratory (Laborarztpraxis Dres. med. Walther, Weindel und Kollegen MVZ GbR, Frankfurt am Main, Germany). Applying the mathematical equations provided by Bikle et al. [41], free and bioavailable 25OHD were calculated based on concentrations of serum albumin, total 25OHD and VDBP.

Patients were assessed for presence or absence of inflammatory disease activity on the basis of serum levels of hsCRP (normal value defined as < 5 mg/L) and fecal calprotectin levels (normal value defined for patients with IBD as < 250 µg/g) [42,43]. High values of either hsCRP or fecal calprotectin, or a combination of both, are considered a sign of inflammatory activity.

### 2.3. Statistical Analysis

Statistical analysis was performed using IBM SPSS version 24.0 and figures were created in Microsoft Excel 2019. The variables were investigated for normal distribution using both visual (histograms, probability plots) and analytical methods (Kolmogorov-Smirnov test). Descriptive analysis was presented as median and range. For comparison of the independent groups, appropriate parametric or non-parametric tests were performed on the basis of a normal distribution using the Student’s *t* or Mann Whitney U tests. For the correlation analyses a Spearman’s test was used due to non-normal distribution of the data. Data were plotted as raw values. Statistical significance was predetermined as *p* < 0.05.

## 3. Results

### 3.1. Study Population

Ultimately, 188 subjects with IBD meeting the inclusion criteria (88 male, 100 female) aged 18–65 years, with a mean age (± SD) of 45.5 ± 14.1 years, were recruited to the study in 2019. The subjects included 84 patients with Crohn’s disease and 104 with ulcerative colitis. In total, 67/188 (36%) of those enrolled had inflammatory disease activity (hsCRP ≥ 5 mg/L and/or fecal calprotectin ≥ 250 µg/g). Mean body mass index was 24.9 ± 5.1 kg/m^2^ for the whole study population and no difference was seen between the inflammatory and noninflammatory groups (*p* = 0.351, Mann Whitney U test). Many patients were on tumor necrosis factor alpha (TNF-α) inhibitor therapy (83/188, 44.1%), while 12.8% of the patients were on antibiotics. The majority of blood samples were taken in the autumn/fall season in both the inflammatory and noninflammatory groups. No significant difference was found according to the season of the blood sample collection (*p* = 0.183, chi square test). Subject characteristics classified according to inflammatory status are described in Table 1.

### 3.2. Laboratory Characteristics

Laboratory characteristics of the subjects with and without active inflammation are presented in Table 2. Overall, as expected, subjects with inflammatory activity had significantly higher leukocyte, erythrocyte sedimentation rate (ESR), hsCRP and fecal calprotectin levels, as well as lower transferrin, transferrin saturation and albumin levels, than patients without inflammatory disease activity. Serum 1,25OH2D and VDBP levels were shown to differ between the inflammatory and noninflammatory groups: patients with inflammatory disease activity had significantly higher serum concentrations of 1,25OH2D (35.0 (16.4–67.3) vs. 18.5 (1.2–51.0), *p* < 0.001) and VDBP (351.2 (252.2–530.6) vs. 330.8 (183.5–560.3), *p* < 0.05) than patients without active inflammation. No differences between the inflammatory and non-inflammatory groups were observed for any of the other vitamin D parameters. For further analysis, correlation coefficients were calculated.

Levels of the different vitamin D metabolites according to the patients’ disease type (Crohn’s disease (CD) versus ulcerative colitis (UC)) are shown in Table 3. No significant differences were identified between the two disease groups for any of the vitamin D markers. A further analysis combining both inflammatory status and type of disease to classify the patients produced similar results; vitamin D markers did not significantly differ between CD and UC patients, regardless of whether inflammation was present.

#### Correlation between Inflammatory Markers and Vitamin D Parameters

**Figure 1:**
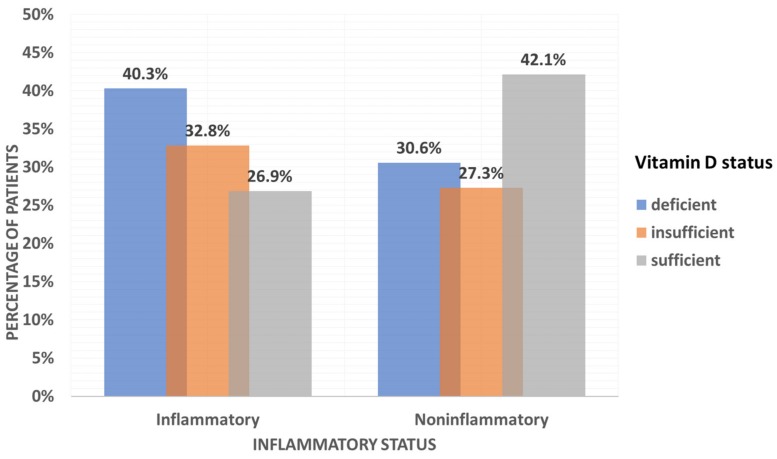
Classification of patients according to vitamin D status and inflammatory activity.

Nonlinear correlations of ESR, albumin, transferrin, hsCRP and fecal calprotectin with selected vitamin D parameters are presented in Table 4. Serum 24,25OH2D levels were negatively correlated with ESR (−0.155, *p* = 0.049), while concentrations of serum 1,25OH2D correlated positively with hsCRP (0.157, *p* = 0.036). Both correlations were weak but significant. Weak positive correlations with serum VDBP levels were found for ESR (0.150, *p* = 0.049), transferrin (0.160, *p* = 0.037) and hsCRP (0.261, *p* < 0.001). The calculated levels of serum free and bioavailable 25OHD showed a weak negative correlation with ESR (−0.165, *p* = 0.031, −0.205, *p* < 0.001, respectively), hsCRP (−0.164, *p* = 0.032, −0.208, *p* < 0.001 respectively) and a moderate negative correlation with fecal calprotectin (−0.377, *p* = 0.028, −0.409, *p* < 0.016, respectively). Serum total 25OHD concentration was the only vitamin D parameter found to have no specific correlation with any of the inflammatory markers (Table 4). There was also no statistically significant difference in serum total 25OHD levels between the inflammatory and non-inflammatory groups (Table 2). When the study population was classified according to vitamin D status using serum 25OHD levels (where vitamin D sufficiency was defined as 30–100 ng/mL, insufficiency as 21–29 ng/mL and deficiency as < 20 ng/mL [40]), the proportion of patients with active inflammation was observed to be higher in the groups with vitamin D deficiency and insufficiency (Figure 1). However, correlations between serum 25OHD and inflammatory parameters were not statistically significant (Table 4).

## 4. Discussion

Due to its higher concentration in blood, relatively longer half-life and better stability compared to other vitamin D metabolites [24,27,44], circulating levels of 25OHD represent the most commonly utilized parameter for the assessment of vitamin D status [24]. Therefore, clinical definitions and recommendations for vitamin D insufficiency/deficiency are based on total 25OHD levels [26]. Consequently, the majority of epidemiologic studies focusing on vitamin D in relation to human health outcomes have used total 25OHD concentrations as a marker [27]. However, 25OHD is less active than some other forms of vitamin D and has to be converted to 1,25OH2D by CYP27B1 (25-hydroxyvitamin D(3)-1alpha-hydroxylase) to achieve its full biologic potency [25,45].

Similar to steroid hormones, the mechanism of action of active 1,25OH2D is mediated through binding to VDR, which regulates genomic (and to some degree nongenomic) intracellular actions of vitamin D [24,45]. During their development or activation, most if not all cells express VDR. Therefore, 1,25OH2D can be synthesized locally in a tissue-specific fashion [8,11,14]. Furthermore, on the basis of recent research, it may be postulated that other vitamin D metabolites and analytes may be useful adjunctive parameters for a more thorough characterization of individual vitamin D status; for example, 24,25OH2D, which might be used to assess catabolism of 25OHD and 1,25OH2D [27,30,46]. Thus, the question arises as to which one of these metabolites, or which combination of metabolites, provides a better representation of vitamin D status in the setting of which physiological/pathophysiological condition. Additionally, accumulating evidence that 25OHD, the traditional marker of vitamin D status, is an acute-phase reactant [34,35,36,37] poses the additional question of whether this is also the case in the setting of chronic inflammation: Can 25OHD be regarded as a reliable biomarker of vitamin D status in patients with chronic inflammatory conditions?

Regarding the use of 1,25OH2D and 24,25OH2D as markers of vitamin D status, our results identified a significant inverse correlation between 1,25OH2D and hsCRP, and a significant positive correlation between 24,25OH2D and ESR. Although the correlation was not strong, the fact that it was significant suggests that these two parameters may be related to—and/or influenced by— inflammation, thus calling into question their accuracy as markers of true vitamin D status in patients with inflammatory disorders. Additionally, given its tight regulation by parathyroid hormone (PTH), calcium and phosphate, its relatively short half-life, and its ability to be synthesized locally, the added benefit of measuring 1,25OH2D remains uncertain [13,27]. An even stronger correlation with PTH has been observed for 24,25OH2D. However, it might be useful as an indicator of tissue-level 1,25OH2D activity [22,27]. While both parameters could be used to gain a better insight into individual vitamin D status, neither 1,25OH2D nor 24,25OH2D seems to be a better sole marker of vitamin D status than 25OHD.

As mentioned above, 25OHD has been discussed as a possible acute-phase reactant. Several studies have investigated the relationship between low serum 25OHD levels and inflammation/inflammatory markers/disease activity in a population of patients with IBD. The results have been inconsistent: whereas many studies show an inverse correlation between 25OHD levels and inflammatory markers and/or disease activity [23,47,48,49,50], numerous other studies have failed to confirm this correlation or relation [51,52,53,54,55]. When the patients from the present study were classified according to serum total 25OHD levels, the proportion of vitamin D-deficient and -insufficient patients with active inflammation was increased compared with the group of patients with normal vitamin D levels. There was, however, no correlation between serum total 25OHD concentrations and any of the available inflammatory markers. This is perhaps explained by the fact that fluctuations in the concentration of CRP are likely to be less pronounced in the context of chronic inflammatory disease than subsequent to an acute-phase response. Therefore, in view of the results from this study in an IBD population, evidence from studies assessing acute-phase response cannot simply be extrapolated to explain the relationship between systemic inflammatory response and serum 25OHD concentrations in patients with chronic inflammatory disease.

Our results suggest that 25OHD currently seems to be the most reliable marker of vitamin D status, even in patients with chronic inflammatory disorders such as IBD. However, doubts remain. Analogous to steroid and thyroid hormones, vitamin D and its hydroxylated metabolites circulate in the blood by binding to a transport protein. The majority of circulating 25OHD is tightly bound to VDBP (85–90%), an α-globulin synthesized by the liver, kidneys and certain other tissues. Approximately 10–15% of serum 25OHD is bound to albumin, while less than 0.1% of 25OHD remains in free circulation [24,27,45]. The sum of albumin-bound and free 25OHD is termed “bioavailable vitamin D”, while the sum of free, albumin-bound and VDPB-bound vitamin D fractions is known as “total vitamin D”. All clinical definitions and recommendations for vitamin D sufficiency are based on total 25OHD [24,27,45]. Nonetheless, several studies have recently suggested that, for certain outcomes (especially relating to bone and calcium metabolism), levels of free or bioavailable 25OHD may be more accurate markers than total 25OHD [24,44]. We therefore considered whether this applies in the presence of chronic inflammation and addressed the question of whether total concentrations of these metabolites, or free levels, can most accurately determine vitamin D status in this case. Interestingly, according to our results, although serum levels of free and bioavailable 25OHD were negatively correlated with ESR, hsCRP and fecal calprotectin, total 25OHD levels were apparently not significantly related to chronic inflammation. It would be exciting to further investigate the implications of our results in future studies, especially concerning the role of VDBP, which we found to be positively correlated with ESR, transferrin and hsCRP. Coming back to the focus of this study, it can be concluded that neither free nor bioavailable 25OHD seems to be an optimal sole marker of vitamin D status in patients with chronic inflammatory disease.

This study has some limitations which should be considered when the results are generalized: Firstly, the study was conducted in a single outpatient unit in patients with different therapy regimens, and without a healthy control group or a group of patients with a chronic, non-inflammatory illness. The cross-sectional design of the research did not allow conclusions to be drawn with regard to cause or effect. In addition, possible effects of potent IBD medications were not evaluated in detail: In both the inflammatory and non-inflammatory groups, approximately 70% of patients were currently receiving treatment with corticosteroids, immunomodulators (e.g., azathioprine, methotrexate) or tumor necrosis factor (TNF)-alpha inhibitors, which are known and intended to reduce chronic inflammation and the bioclinical parameters thereof, and may also affect vitamin D metabolism. However, the scope of the study did not allow the identification of specific effects of these medications, and since the distribution differed only insignificantly between the groups, their overall effect on the study results were assumed to be negligible. The study design also precluded the assessment of possible changes in total 25OHD levels in parallel to clinical improvement of the disease. Additionally, since PTH, calcium or phosphate levels were not determined, that might have provided valuable additional information in the interpretation of the findings, the question of whether 25OHD acts like an acute phase reactant in IBD remains open. While fecal calprotectin and hsCRP levels are recognized to be accurate markers of inflammation in general, the fact that clinical disease activity indices were not used as supplementary indicators of inflammatory disease activity may be regarded as a weakness of the study. Levels of free and bioavailable 25OHD were calculated rather than specifically measured, and there is some evidence in the existing literature that calculated levels might not always perfectly reflect actual measured levels. However, to the best of our knowledge, this study in patients with IBD is the very first to investigate the best marker of vitamin D status in patients with chronic inflammatory disease, and an important first step to further, deeper exploration. On the basis of our results, we suggest that direct quantification of free vitamin D metabolites might be a promising area for future research.

## 5. Conclusions

In conclusion, all of the vitamin D markers assessed in this study, including serum levels of 1,25OH2D, 24,25OH2D, calculated free and bioavailable 25OHD and VDBP were found to be influenced by the presence of inflammation, with the exception of total 25OHD levels. Thus, the traditional parameter, total 25OHD, still appears to be a reliable marker of vitamin D status in IBD patients with evidence of inflammation.

## Figures and Tables

**Table 1:** Patient characteristics according to presence or absence of inflammation.

	Inflammatory (hsCRP ≥ 5 mg/L and/or fecal calprotectin ≥ 250µg/g, *n* = 67)	Noninflammatory (hsCRP < 5 mg/L and fecal calprotectin < 250µg/g, *n* = 121)	*p*_1_	*p*_2_
N (♀)	67 (30)	121 (70)	0.108	-
Age (mean ± SD)	43.4 ± 14.2	45.4 ± 14.3	-	0.351
BMI (kg/m^2^, mean ± SD)	25.6 ± 5.4	24.6 ± 4.9	-	0.308
Weight (kg, mean ± SD)	76.1 ± 15.5	72.1 ± 17.8	-	0.171
Height (cm, mean ± SD)	171.7 ± 10.0	176.9 ± 9.8	-	0.310
**Disease type, *n* (%)**				
Crohn’s disease	30 (44.8%)	54 (44.6%)	0.676	-
Ulcerative colitis	37 (55.2%)	67 (55.4%)		
**Medication, *n* (%)**				
5-ASA or no treatment	11 (16.4%)	27 (22.3%)	0.335	-
Immunomodulator	14 (20.9%)	17 (14.0%)	0.226	
Anti TNF	31 (46.3%)	52 (43.0%)	0.663	
Corticosteroids	6 (9.0%)	15 (12.4%)	0.473	
Antibiotics	11 (16.4%)	13 (10.7%)	0.264	
**Seasons of blood samples, *n* (%)**				
Winter-Spring	31 (46.3)	57 (47.1)	0.663	-
Summer-Autumn/Fall	36 (53.7)	64 (52.9)		

hsCRP: high-sensitivity C-reactive protein, SD: standard deviation, BMI: body mass index, TNF: tumor necrosis factor, *p*_1:_ chi square test, *p*_2:_ independent samples test.

**Table 2:** Comparison of laboratory parameters in the inflammatory and noninflammatory groups.

Laboratory parameters	Inflammatory (hsCRP ≥ 5 mg/L and/or fecal calprotectin ≥ 250 µg/g, *n* = 67) median (min-max)	Noninflammatory (hsCRP < 5 mg/L and fecal calprotectin < 250 µg/g, *n* = 121) median (min-max)	*p*
Erythrocytes	4.8 (3.4–6.2)	4.6 (4.6–3.3)	0.085
Haemoglobin (g/dL)	14.0 (8.0–16.5)	14.3 (14.3–9.5)	0.994
Haematocrit (%)	41.7 (25.9–417.0)	41.3 (30.0–50.9)	0.552
Leukocytes	8.3 (4.4–16.4)	7.2 (4.0–16.4)	<0.001**
Transferrin (mg/dL)	251.0 (144.0–353.0)	263.0 (167.0–404.0)	0.053
TSAT (%)	17.7 (5.3–47.1)	25.7 (6.0–143.0)	<0.001**
Albumin (g/L)	43.0 (31.0–52.0)	45.0 (28.0–51.0)	0.014*
ESR (mm/h)	14.0 (2.0–63.0)	4.0 (2.0–46.0)	<0.001**
hsCRP (mg/L)	8.3 (1.0–20.8)	1.2 (1.0–4.8)	<0.001**
Fecal calprotectin (µg/g)	245.0 (69.0–718.0)	100.0 (55.0–245.0)	<0.001**
**Vitamin D markers**			
25OHD (ng/mL)	27.1 (6.8–65.5)	26.9 (5.0–74.3)	0.707
24,25OH2D (ng/mL)	2.1 (0.4–9.6)	2.3 (0.1–17.9)	0.552
1,25OH2D (pg/mL)	35.0 (16.4–67.3)	28.5 (1.2–51.0)	<0.001**
VDBP (mg/dL)	351.1 (252.2–530.6)	330.9 (183.5–560.3)	0.021*
Free 25OHD (pg/L)	5.8 (1.3–17.9)	6.1 (1.0–51.4)	0.469
Bioavailable 25OHD (ng/L)	2.4 (0.1–7.3)	2.5 (0.5–19.5)	0.325

ESR: erythrocyte sedimentation rate, TSAT: transferrin saturation, VDBP: vitamin D binding protein, *p*: Mann Whitney U test significance; * *p* < 0.05, ** *p* < 0.001.

**Table 3:** Comparison of vitamin D markers in patients with Crohn’s disease (CD) versus ulcerative colitis (UC).

Vitamin D markers	Crohn’s Disease (*n* = 84) median (min-max)	Ulcerative Colitis (*n* = 104) median (min-max)	*p*
25OHD (ng/mL)	26.4 (5.0–74.4)	26.0 (6.8–63.5)	0.707
24,25OH2D (ng/mL)	2.1 (0.1–17.9)	2.1 (0.1–7.7)	0.552
1,25OH2D (pg/mL)	31.0 (1.2–67.3)	35.7 (3.3–62.0)	0.051
VDBP (mg/dL)	344.2 (183.5–556.3)	337.2 (248.0–560.3)	0.121
Free 25OHD (pg/L)	5.9 (1.0–51.4)	5.8 (1.3–13.5)	0.325
Bioavailable 25OHD (ng/L)	2.4 (0.5–19.5)	2.4 (0.1–5.8)	0.469

*p*: Mann Whitney U test significance.

**Table 4:** Nonlinear correlations of ESR, albumin, transferrin, HsCRP and fecal calprotectin with selected vitamin D parameters.

Selected biomarker	Vitamin D parameter	Spearman’s rho	*p*
**ESR (mm/h) vs.**	25OHD (ng/mL)	−0.080	0.286
	24,25OH2D (ng/mL)	−0.155	0.049*
	1,25OH2D (pg/mL)	0.069	0.358
	VDBP (mg/dL)	0.150	0.049*
	Free 25OHD (pg/L)	−0.165	0.031*
	Bioavailable 25OHD (ng/L)	−0.205	<0.001**
**Albumin (g/L) vs.**	25OHD (ng/mL)	−0.013	0.863
	24,25OH2D (ng/mL)	0.047	0.539
	1,25OH2D (pg/mL)	−0.044	0.558
	VDBP (mg/dL)	0.028	0.706
	Free 25OHD (pg/L)	−0.024	0.745
	Bioavailable 25OHD (ng/L)	0.129	0.086
**Transferrin (mg/dL) vs.**	25OHD (ng/mL)	0.039	0.607
	24,25OH2D (ng/mL)	0.067	0.379
	1,25OH2D (pg/mL)	0.133	0.078
	VDBP (mg/dL)	0.160	0.037*
	Free 25OHD (pg/L)	−0.012	0.881
	Bioavailable 25OHD (ng/L)	−0.003	0.970
**HsCRP (mg/L) vs.**	25OHD (ng/mL)	−0.052	0.498
	24,25OH2D (ng/mL)	−0.080	0.295
	1,25OH2D (pg/mL)	0.157	0.036*
	VDBP (mg/dL)	0.261	<0.001**
	Free 25OHD (pg/L)	−0.164	0.032*
	Bioavailable 25OHD (ng/L)	−0.208	<0.001**
**Fecal calprotectin (µg/g) vs.**	25OHD (ng/mL)	−0.315	0.065
	24,25OH2D (ng/mL)	−0.264	0.120
	1,25OH2D (pg/mL)	0.321	0.056
	VDBP (mg/dL)	0.034	0.847
	Free 25OHD (pg/L)	−0.377	0.028*
	Bioavailable 25OHD (ng/L)	−0.409	0.016*

vs.: versus. * *p* < 0.05, ** *p* < 0.001.

## Abbreviations

1,25OH2D	1,25-dihydroxyvitamin D
24,25OH2D	dihydroxycholecalciferol
25OHD	25-hydroxyvitamin D
CD	Crohn’s disease
ESR	erythrocyte sedimentation rate
hsCRP	high sensitivity C-reactive protein
IBD	inflammatory bowel disease
PTH	parathyroid hormone
TSAT	transferrin saturation
UC	ulcerative colitis
VDBP	vitamin D binding protein
VDR	vitamin D receptor
